# Weather in two climatic regions shapes the diversity and drives the structure of fungal endophytic community of bilberry (*Vaccinium myrtillus* L.) fruit

**DOI:** 10.1186/s40793-024-00551-y

**Published:** 2024-01-22

**Authors:** Minh-Phuong Nguyen, Kaisa Lehosmaa, Katalin Toth, Janne J. Koskimäki, Hely Häggman, Anna Maria Pirttilä

**Affiliations:** 1 Ecology and Genetics Research Unit, University of Oulu, P.O. Box 3000, 90014 Oulu, Finland; 2 Inari Agriculture Nv, Industriepark Zwijnaarde 7a, 9052 Ghent, Belgium

**Keywords:** Fruit microbiology, Fungal endophyte, Climate, Weather conditions, Growth season, Diversity, Community structure, Bilberry

## Abstract

**Background:**

Bilberry (*Vaccinium myrtillus* L.) is one of the most important economic and natural resources in Northern Europe. Despite its importance, the endophytic fungal community of the fruits has rarely been investigated. Biogeographic patterns and determinants of the fungal diversity in the bilberry fruit are poorly understood, albeit fungal endophytes can have a close relationship with the host plants. Here, we investigated the effect of climatic regions, and their weather conditions within growth season and soil properties on fungal endophytic communities of bilberry fruits collected from northern and southern regions of Finland using high-throughput sequencing technology targeting the internal transcribed spacer 2 ribosomal DNA region for fungi.

**Results:**

Species richness and beta diversity (variation in community structure) were higher in the southern compared to the studied northern region. The weather condition of the growth season drove both fungal richness and community structure. Furthermore, abundance of the genera *Venturia*, *Cladosporium*, and *Podosphaera* was influenced by the weather, being different between the south and north regions.

**Conclusions:**

We conclude that diversity and assembly structure of the fungal endophytes in bilberry fruits follow similar patterns as for foliar fungal endophytes, being shaped by various environmental factors, such as the climate and surrounding vegetation.

**Supplementary Information:**

The online version contains supplementary material available at 10.1186/s40793-024-00551-y.

## Background

Bilberry (*Vaccinium myrtillus* L.) is one of the economically important natural resources in Northern Europe [1, 2] and a well-known functional and therapeutic food [3, 4]. Bilberries are widely picked for domestic use and commercial sale [1, 2]. The fruits are used in the food industry (e.g., juices, jams), and are a potential source for extraction of bioactive compounds, such as anthocyanins and waxes [5]. A daily diet including bilberries has a positive impact on human health, as bilberries are a rich natural source of phenolic compounds (containing up to 15 kinds of anthocyanins) [1, 6, 7]. The anthocyanins have great health-beneficial properties, such as antioxidant, anticancer, cardioprotection, neuroprotection, diabetes protection, and eyesight improvement properties [1, 3], which make bilberries a valuable resource in northern countries.

Healthy plants are hosts for a diverse community of microbes both on the surface (epiphytes) and inside the plant tissues (endophytes) [8]. Endophytes, which are mainly bacteria and fungi, take shelter and nutrition from the host plants and simultaneously benefit the hosts [9]. For example, fungal endophytes can produce growth-inhibitory compounds against plant pathogens and herbivores [10, 11], or induce the biosynthesis and accumulation of phenolic acids in the host [12]. Fungal endophytes have also been shown to increase the host’s tolerance towards abiotic stress, such as cold [13], drought and salt by enhancing the antioxidant levels in the plant [14]. Therefore, endophytes are considered to have an intimate relationship with their hosts.

However, the lifestyle of fungal endophytes is still, to a great deal, a mystery. The ecological function of endophytes depends on their interaction with the host plant [9], the host genotype and fitness [15], and the endophytic community structure of the host [16]. Understanding how the endophytic community of fruits is formed may help to develop traceability analyses of fruits [17], and management strategies against plant diseases by improving plant growth or quality of the fruits, as well as by controlling postharvest diseases [18]. Therefore, it is essential to investigate the fungal endophytic community and the factors shaping the fungal community structure.

Comprehensive studies on fungal community structures and their main drivers are scarce in fruits compared to other plant parts. A few studies have shown that the diversity of fungal communities in fruits is driven by the host species [19, 20], and other plant organs [21]. A study on grapevine showed that the fungal community was different between fruits and other tissues and habitats (e.g., leaves, roots, and soil communities), and the grape berry microbiota significantly correlated with weather parameters [22]. However, that study did not separate between epiphytic and endophytic communities but treated them as one, which explains the effect of weather, as epiphytes are more readily exposed to environmental conditions [23]. Moreover, studies on fruits of wild plant species are rare while the main focus of interest has been on commercial fruits, such as grape berries [24], cranberry ovary [25], coffee berries [26], apple and pear [27], blueberry [28, 29], and tomato [21]. Therefore, the need for comprehensive studies on the fungal endophytic community of wild fruits is urgent.

Bilberry is an excellent subject to study the effect of climatic conditions and other environmental factors on the diversity of endophytic communities in wild berry fruits because it lives in harsh environmental conditions expanding through latitudes. With that in mind, we investigated the fungal communities of bilberry fruits collected from two different climatic areas corresponding to the northern and southern regions of Finland (so-called north and south regions) to answer the following questions: (1) Does the diversity of fungal communities differ between north and south regions; (2) Are the differences of diversity driven by above (weather conditions of the growth season) or below ground (soil) factors; (3) Do the driving factors differ for each region; (4) Which fungal genera are driven by the examined environmental variables. Since fruits are similar to deciduous leaves in terms of being formed during the growth season and then going through abscission, we expected that fungal endophytes of fruits might follow some of the patterns found for foliar fungal endophytes. We expected the fungal endophytes of fruits to be horizontally transmitted from the surrounding environment, similar to the endophytes of leaves [30]. Due to the possible horizontal transmission, the factors affecting the diversity of fungal communities in fruits could be diverse. For example, the colonization of fungal endophytes in cacao leaves is driven by the amount of airborne inoculum and the high relative humidity or the wetness of leaf surfaces [30] whereas it is not affected by leaf toughness or leaf chemistry. The diversity of fungal endophytes of leaves also follows a strong latitudinal gradient but is not affected by annual precipitation [31]. Overall, our study provides insights into the environmental factors shaping the fungal community of bilberry, in particular, and wild berry fruits, in general.

## Methods

### Study site selection

We focused on government-owned national parks and protected areas in Finland to minimize human impact. Sampling sites were selected based on open geographic information systems (GIS) data provided by the Finnish Environment Institute (SYKE) and spatial analysis were conducted with R software environment version 4.2.2 [32] using packages: *raster* [33], *rgdal* [34], *sf* [35], *dplyr* [36], and *ggplot2* [37]. Specifically, we generated 100 random points for each polygon of the national parks in the GIS-data using *st_sample* function of package *sf*. Then we extracted the information of vegetation types for these random points based on the Corine dataset (2018, grid size 20 m × 20 m), which classifies the land use into different categories. The national parks which contained at least 50 random points classified as “coniferous forest on mineral soil” were selected for the next step. We focused on “coniferous forest on mineral soil” because bilberry is one of the most common coniferous forest dwarf shrubs in Finland, and this category of coniferous forests is the most common in Finland.

**Fig. 1:**
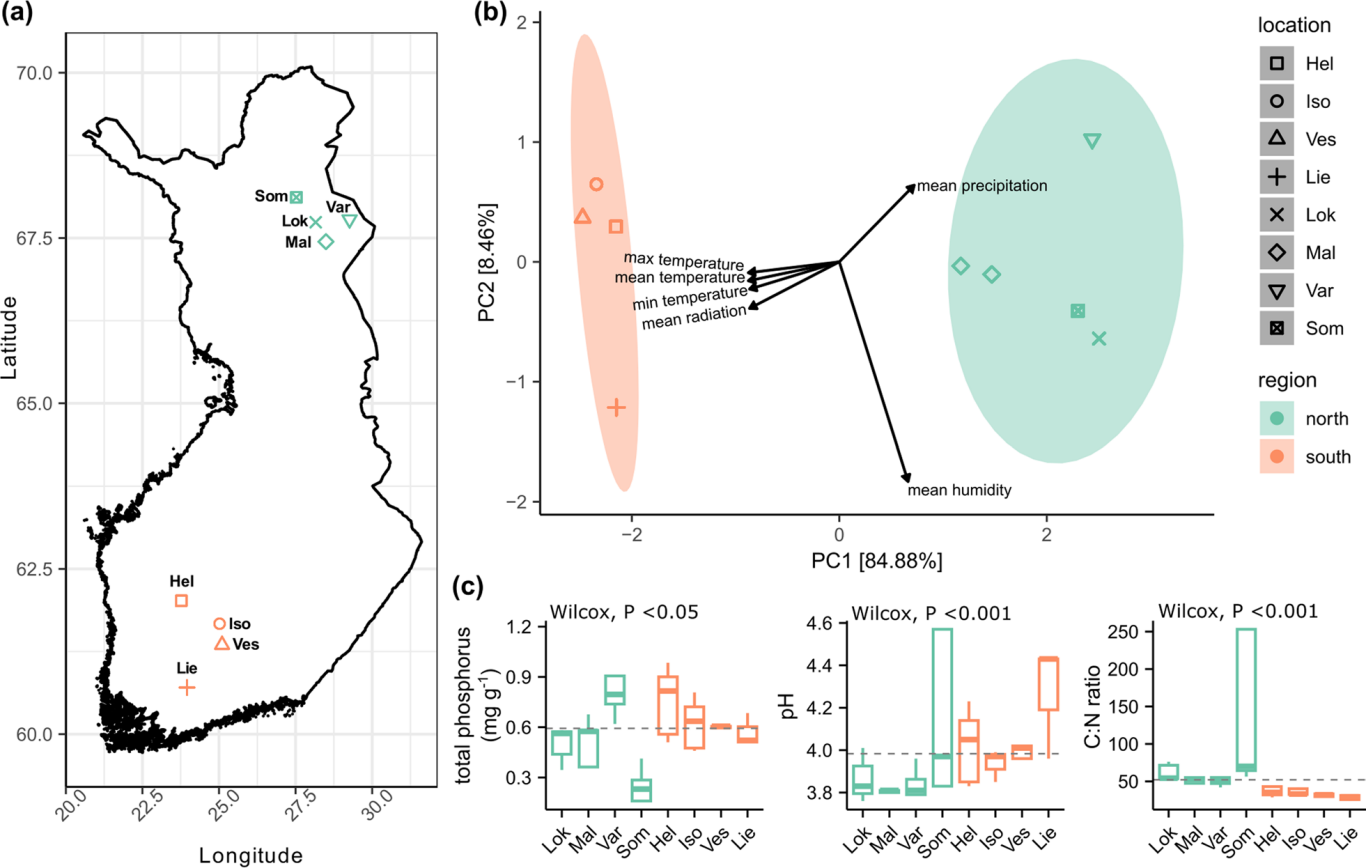
Sampling sites and the examined environmental variables. **a** A map of the sampling sites of the study, **b** a PCA ordination of the weather variables grouped by regions, and **c** boxplots of the soil variables which include the Wilcoxon test’s results for the soil variables between the north and south regions. **a** The sampling sites were selected close to national parks. Som - Sompio Strict Nature Reserve, Var - Värriö Strict Nature Reserve, Lok - Lokka, Mal - Maltio Strict Nature Reserve, Hel - Helvetinjärvi national park, Iso - Isojärvi national park, Ves - Vesijako Strict Nature Reserve, Lie - Liesjärvi national park. **b** The PCA ordination shows the distance between sites (scaling 1), and the ellipse denotes a 95% confidence interval. **c** Dash lines indicate the mean value of the variables. **a**–**c** The sites of the same color belong to the same climatic region

Finland is located between latitude 60°N and 70°N and belongs to the subarctic or boreal climate zone. Therefore, to ensure that we have sampling sites from two regions that have distinguishing climatic parameters, we extracted the real time weather parameters from the ERA5-Land dataset, which is a reanalysis dataset providing an accurate description of the climate in the past [38], and grouped the selected national parks from the previous step based on the weather parameters with *hclust* function of vegan package [39]. The selected weather parameters included 2 m temperature (temperature of air at 2 m above the surface of land, sea or in-land waters), surface net solar radiation (amount of solar radiation reaching the surface of the Earth minus the amount reflected by the Earth's surface), and total precipitation for every hour in the period of 1 August 2020 to 30 April 2021. From each climate cluster, we selected three to four national parks as sampling locations. Värriö (Var), Maltio (Mal), Sompio (Som) Strict Nature Reserve areas and Lokka (Lok, which is close to a natural reserve area) were selected for the north region. Helvetinjärvi (Hel), Isojärvi (Iso), Liesjärvi (Lie) national parks and Vesijako (Ves) Strict Nature Reserve were selected for the south (Fig. 1a).

### Sample collection

At each sampling location (i.e., national parks), four different bilberry clones (i.e., mother plants) were selected (so-called clones A, B, C, D). Four biological replicates were picked from each bilberry clone. The samples were named after their location and clone; for example, Ves-D1 was the first sample collected at clone D of location Vesijako. The total number of samples for each climatic region was 64. Bilberry samples were collected in 50-ml Falcon tubes and kept at + 4 °C until they were brought to the laboratory for surface sterilization (maximum three days since the day of sampling due to the transportation). All samples and their metadata were in the Additional file 1: Table S1.

Simultaneously with the berry collecting, we also sampled topsoil from the area of each bilberry clone. A metal soil drill was bored into the soil down to the depth of 15 cm. Four soil cores for each bilberry clone were pooled together. Only the organic layer (humus) was pooled while the litter and top mineral soil were removed. The soil samples were kept at + 4 °C for transportation and stored at − 20 °C for further analysis.

### Sample preparation

The berry samples were surface sterilized to remove epiphytes and contaminants on the skin as earlier [19] with some modifications. We used 2% calcium hypochlorite instead of 3% solution because the berries became delicate under the summer heat and transportation. Surface-sterilized samples were kept at − 20 °C until they were freeze-dried.

Twenty berries for each sample were pooled and freeze-dried using a vacuum freeze-dryer for 48 h until the majority of water content was removed from the fruits for combining the berries as one sample. The freeze-dried samples were ground into a fine powder by mortar and pestle on a liquid nitrogen bath. An aseptic condition was maintained when working with the samples.

### Molecular analyses and bioinformatics

DNA was extracted from 65 mg of each freeze-dried powder using the protocol of Toth et al. (in preparation) with QIAamp Fast DNA Stool Mini kit (Qiagen). The DNA extracts were diluted to the concentration of 40 ng µl^−1^ for PCR amplification, and 20 µl of the DNA were treated with 5 µg of RNase A (10 mg ml^−1^, Thermo Fisher Scientific) for one hour at 37 °C.

The fungal internal transcribed spacer 2 (ITS2) ribosomal DNA region was amplified in a 20 µl reaction containing 1X Phusion GC buffer, 0.2 µM dNTP, 5% DMSO, 10 µg BSA (New England BioLabs), 0.5 µM of each fungi-specific primer fITS7 (5′-GTGARTCATCGAATCTTTG-3′) [40] and ITS4R (5′-TCCTCCGCTTATTGATATGC-3′) [41], 0.4 U of Phusion High-Fidelity DNA polymerase (Thermo Fisher Scientific), and 200 ng of RNase-treated DNA template. A higher quantity of DNA was used because the amount of endophytic DNA is only a fraction of the total DNA. The PCR program conditions consisted of an initial denaturation of 98 °C for 3 min, followed by 23 cycles of 98 °C for 10 s, 55 °C for 20 s, and 72 °C for 30 s, and a final extension of 72 °C for 7 min. The PCR amplification was done in triplicate for each sample and then pooled. Successful PCR amplification was confirmed by agarose gel electrophoresis of pooled reactions. The PCR products were sent to FIMM Genomics NGS Sequencing Unit at University of Helsinki for the ITS2 amplicon sequencing using fITS7/ITS4R primers on an Illumina MiSeq (300 bp paired-end reads). We also included PCR products from two identical replicates of our custom fungal mock community and negative controls from the DNA extraction and PCR amplification steps in the sequencing.

The reads were demultiplexed at the sequencing center (FIMM). Raw data with 13,456,669 paired reads were processed with QIIME2 pipeline version 2021.11 [42] following the procedure for fungal ITS by Nguyen [43] with some modifications. First, we trimmed primers and used DADA2’s default parameters to truncate forward reads and reverse reads at the position of 275 and 185 bp, respectively, and to denoise and merge paired reads. We clustered the amplicon sequence variants (ASVs) into operational taxonomic units (OTUs) with VSEARCH at the similar threshold of 97%. Then we used ITSx software 1.1.3 to extract the ITS2 region from the sequences [44]. The extracted ITS2 sequences were taxonomically classified in QIIME2 using the UNITE dynamic database 8.3 (released on 10 May 2021) [45]. We kept only the OTUs which gave an 85% match length in the BLAST search. After this, 225,665 fungal ITS2 reads which belonged to 166 OTUs from 127 samples (including mock and negative control samples) were retained. Examining the taxonomic profile of the mock community confirmed the suitability of the procedure for our data.

We used the R package *phyloseq* to handle the processed dataset [46]. We filtered the OTU table by subtracting the reads of the negative controls from the samples [47], and kept the OTUs which were classified to at least the phylum level. We retained the OTUs having at least 5 reads per sample and samples with at least 20 reads [48]. The final processed dataset contained 179,353 reads consisting of 116 OTUs from 113 samples. The median number of reads was 252 and the sparsity ratio was 0.95.

### Soil data

We analyzed pH, total carbon (C, mg g^−1^), nitrogen (N, mg g^−1^) and total phosphorus (TP, mg g^−1^) content from the soil samples. For pH, 20 ml of soil was shaken in 50 ml milli-Q water for one hour, then the mixture was allowed to stand for at least 30 min before analyzing. The supernatant was used for the measurement of pH (pH-meter CG832 Schott Geräte).

To measure total phosphorus, the soil samples were dried at + 40 °C for at least 24 h, then ground into a fine powder by 1.5-cm diameter metal beads in a Retsch MM 301 mixer mill and dried again at + 40 °C for 2 h. The total phosphorus was analyzed by decomposing 0.5 g dried ground soil in 5 ml nitric acid and 2 ml hydrochloric acid in a Microwave sample preparation system (Programme PAAR005H, Perkin Elmer Paar Physica, Multiwave). Afterwards, the sample was diluted with 25 ml milli-Q water. One ml from the dilution was mixed with 5 ml of a combined reagent (1.5% (w/v) ascorbic acid, 2%(w/v) ammonium molybdate, 4.5 M H_2_SO_4_, and 0.05% (w/v) antimony potassium tartrate), then milli-Q water was added to the final volume of 50 ml. The final solution was used for the phosphorus analysis by a UV–visible spectrophotometer (Shimadzu UV-1700). The concentration of phosphorus (mg g^−1^) was calculated based on the formula: ${\text{C}} = \frac{{{\text{C}}^{\prime } \cdot {\text{V}} \cdot {\text{df}}}}{{\text{W}}}$ with C—concentration (mg g^−1^), C′—concentration given by the spectrophotometer (mg l^−1^), V*—*total volume (l), df—dilution factor, W—weight of starting material (g).

For the analysis of carbon and nitrogen contents, seven to eight mg of the dried ground soil was weighed into a tin capsule and analyzed with a FlashSmart CHNS/O Elemental Analyzer (Thermo Fisher Scientific, Bremen, Germany).

### Weather and topographic data

The daily weather data of Finland for the year 2021 in the GeoTIFF format were downloaded from the database of the Finnish Meteorological Institute via the spatial data download service Paituli (https://paituli.csc.fi/). Data from the start of the bilberry growth season till the last sampling date (1 April 2021–5 August 2021) was extracted. The elevation model dataset (grid size 10 m × 10 m), which depicts the elevation of the ground surface in relation to the sea level, was downloaded from the National Land Survey of Finland via Paituli. The information was extracted for each sampling site with a buffer of 20 m. The vegetation type was extracted from the Corine dataset. Due to the difficulty of finding bilberries, we had to shift our sampling sites (clones) from the original plan. As a result, 27 out of 32 sampling clones were classified as “coniferous forest on mineral soil” as planned, and five remaining sites were classified with different vegetation types: mixed forest on mineral soil (Iso-C, Iso-D, Lie-A), mixed forest on peatland (Lie-B), and peatland (Mal-D) (Additional file 1: Table S1). Different vegetation types were considered in modelling.

### Statistical analysis

All analyses were conducted in the R environment, and the packages *phyloseq*, *vegan*, *glmmTmB* [49], *DHARMa* [50], *MuMIn* [51], *microbiome* [52], *microeco* [53], *dplyr*, ALDEx2 [54] and *ggpubr* [55] were used.

We removed Ves-D3 from our dataset, because the sample is the only representative of the clone Ves-D. The remaining dataset included 112 samples and had two to four samples for each bilberry clone.

Differences in soil factors between the north and south were tested with non-parametric Wilcoxon test. The correlation between environmental variables was investigated with the function *cor* (Additional file 2: Fig. S1). Principal component analysis (PCA) was conducted for all weather variables of the growth season using the *prcom* function because they were highly correlated (r Pearson > 0.6), and the PC1 axis (so-called PC1) was used for further analysis because it explained 85% of the variation (Fig. 1b) [56]. The altitude highly correlated with the weather variables; therefore, altitude was removed from further analysis. The quantities of C and N were highly correlated, therefore C:N ratio was calculated. C:N ratio, total phosphorus (TP) and pH were included in further analysis. All included variables were scaled because they were measured in different units. Definitions of all environmental variables are explained in Additional file 2: Table S2.

We calculated the number of observed species (richness) and investigated if the species richness differed by climatic regions by fitting a generalized linear mixed model (GLMM) of the species richness as a function of climatic regions, sequencing depth as an offset and “clone” as a nested random effect in “region” using the *glmmTmB* function and family Poisson. Model validation was verified with the package *DHARMa*. To investigate which environmental variables affected the species richness we also fitted a similar GLMM model of the richness as a function of the environmental variables. We first included all potential variables (PC1, pH, C:N ratio and TP) in the full model. Then we did the backward model selection to remove nonsignificant variables to find the most simple and adequate model. Starting from the full model, we removed non-significant variables one by one based on their *P* values. The non-significant variable that had the largest *P* value was removed first. Second-order Akaike Information Criterion (AICc) values were calculated by function *AICc* of the package *MuMIn*. The final model was selected based on the model validation and AICc values. We also did the modeling for each region separately to check if they had their own trends. All models included in the model selection and their AICc values were in Additional file 2: Table S3.

We applied a robust Aitchison dissimilarity metric (so-called dRAI) on the OTU table using the *vegan* package. The method was chosen since it effectively accounted for data compositionality and sparsity [48]. To assess beta diversity (variation in community structure), we used the function *betadisper* in *vegan* to generate distance-to-centroid values (i.e., multivariate dispersion) for the dRAI metric grouped by region. The function *permutest* was used to test if one group is more dispersed than the other, taking into account our study design. To test if the examined environmental variables or the fungal richness affect the beta diversity, we fitted separate GLMM models of square root distance-to-centroid values as a function of environmental variables or fungal richness using family Gaussian and selected the best models for each case.

Assembly structure between the north and south regions were tested with Permutational Multivariate Analysis of Variance (PERMANOVA) [57] on the dRAI matrix with region as the fixed effect term using *adonis2* function. We specified “strata = location” to account for our study design. We tested whether any environmental variables explained fungal community composition by using distance-based redundancy analysis (dbRDA) taking the dRAI matrix as the response variable and PC1 and soil variables as the explanatory variables using the function *dbrda* of the *vegan* package. We specified “Condition (location)” in the model to take our study design into account. Significance of the model and the marginal effect of the terms were tested by permutation tests (9999 permutations), taking into account the structure of our data. Similarly, we also applied dbRDA for each region to discover the importance of the tested environmental variables on the structure of the fungal community in each region. Finally, the reduced dbRDA models containing only the terms that had significant marginal effect were produced and visualized.

Relative abundance of the fungal community was visualized by stacked bar plots and heatmaps. Shared and unique OTUs between the north and south regions were illustrated by a Venn diagram using the package *microeco*. Differential abundance analysis between the two regions was carried out at the genus level of the fungal data using package *ALDEx2*. We first merged the reads at the genus level using the *tax_glom* function of the *phyloseq* package and filtered out taxa with less than 10% prevalence. The significant taxa after the differential abundance analysis were selected based on the Wilcoxon *P*-adjusted values implemented in ALDEx2. To examine the effect of PC1 and soil variables on the abundance of these taxa, we fitted GLMM models to the absolute abundance of the significant taxa with either using the family Poisson or negative binomial and selected the best model for each genus with backward selection. The correlation between the genus’ abundance and the environmental variable’s gradient which had a significant effect in the GLMM models were visualized by fitting their vectors on the Principal Coordinate Analysis (PCoA) ordination of the fungal data using the function *envfit* of *vegan* package [58].

## Results

### Fungal richness and beta diversity were higher in the south region

**Fig. 2:**
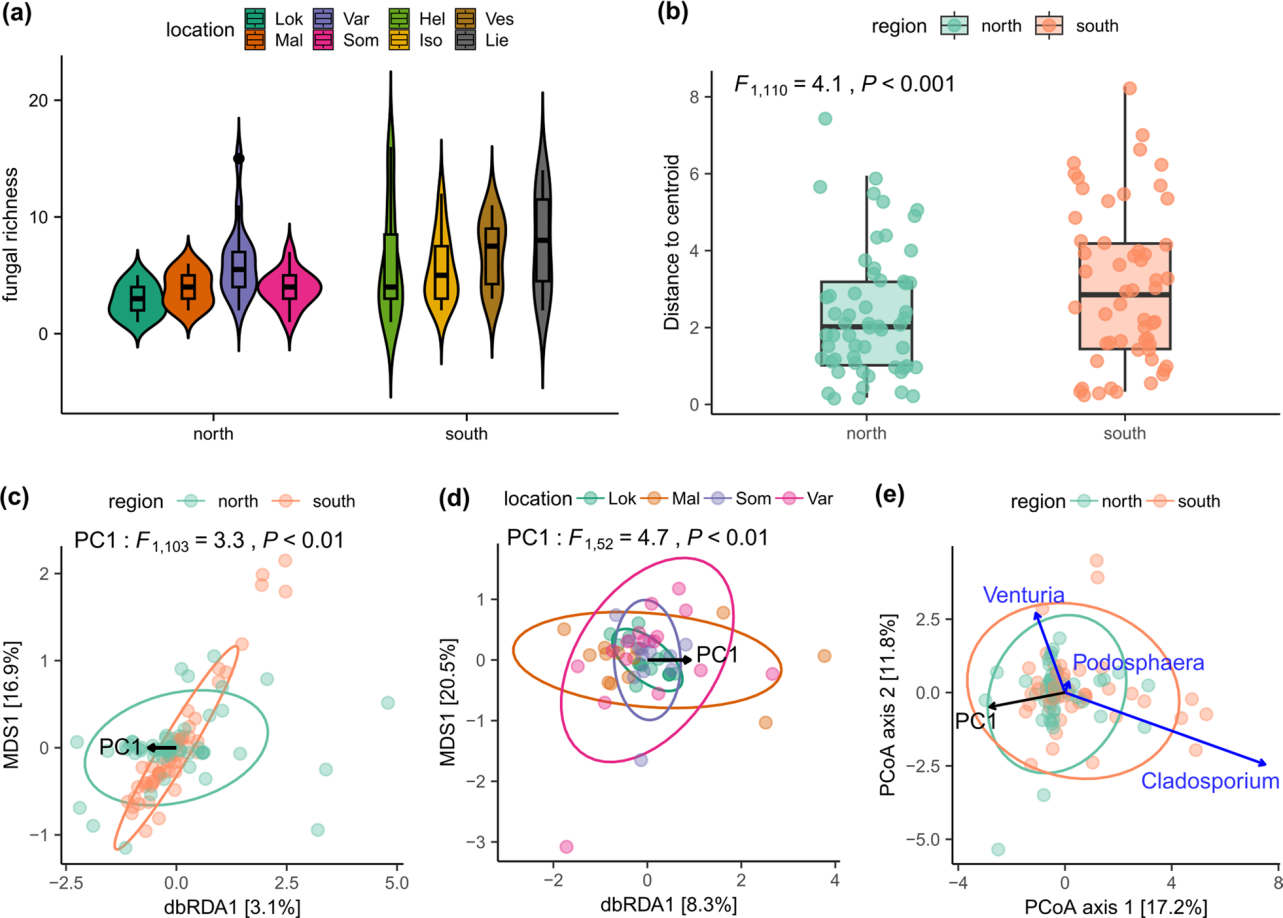
Diversity of the fungal community. **a** Fungal richness, **b** multivariate dispersion, **c** dbRDA plots visualizing the PC1 variable explaining the fungal community assemblages for the whole dataset or **d** for the north region, and **e** a PCoA plot depicting the correlation between the abundance of the genera of interest and the gradient of the PC1 variable. Ellipses denote a confidence interval of 75% (in **c** and **d**) and 95% (in **e**)

The fungal richness in the south was significantly higher compared to the north region (Fig. 2a, Additional file 2: Table S4). Among the tested environmental variables, PC1 significantly influenced the fungal richness whereas TP, C:N ratio and pH had no effect (Additional file 2: Table S5).

Multivariate dispersion was larger for the south compared to the north region (*F*_1,110_ = 4.1, *P* < 0.001, Fig. 2b), and it was driven by the fungal richness (Additional file 2: Table S6) but not by PC1 or soil variables.

### Community structure was driven by the weather conditions

The community composition did not clearly differ between the two regions (PERMANOVA, *F*_1,110_ = 2.02, *P* = 1). However, the dbRDA model with PC1 and soil variables was significant (*F*_4,100_ = 1.6, *P* < 0.01) although it explained only a small proportion of the community variation (adjusted R^2^ = 2.3%). Among the tested explanatory variables, the PC1 affected the community structure (*F*_1,100_ = 2.87, *P* < 0.01, Additional file 2: Table S7), whereas the soil variables did not. The reduced dbRDA model with only PC1 as the explanatory variable for the whole dataset explained 2% of the community variation (Fig. 2c). The full dbRDA model for the north region with all tested explanatory variables was significant (*F*_4,49_ = 1.97, *P* < 0.01), but only the PC1 had a significant marginal effect (*F*_1,49_ = 3.26, *P* < 0.05, Additional file 2: Table S7), and this model explained 6.3% of the total variation (adjusted R^2^ = 6.3%). The reduced model for the north explained 6% of the north community variation (Fig. 2d).

### Fungal community profiles differed between climatic regions

The phylum *Ascomycota* was dominant in both southern and northern samples (in average 93% and 99%, respectively). The mean relative abundance of *Basidiomycota* was higher in the south compared to the north (7.4% vs. 0.5%), due to its high abundance in the locations Iso, Lie and Ves of the south (6%, 12%, 12%, respectively). The phylum *Mucoromycota* was found only in the samples of the location Lok of the north (1%), whereas *Olpidiomycota* was only present in the Ves samples of the south (< 1% abundance).

**Fig. 3:**
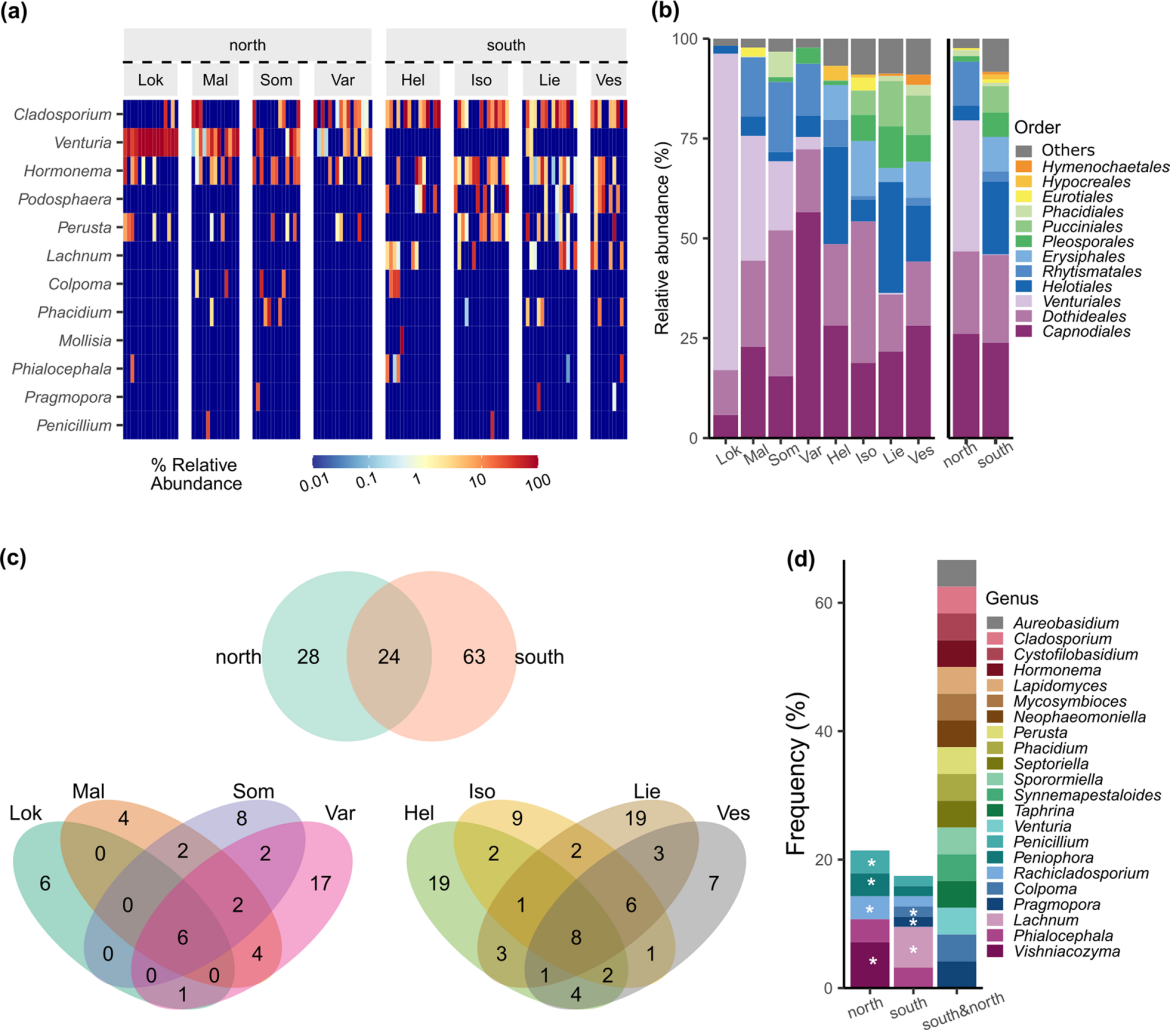
Fungal community profile in the two regions. **a** Fungal community profile in the two regions demonstrated by heatmap at the genus level and **b** stacked bar plot at the order level, **c** Venn diagrams of the shared OTUs between regions and between locations for each region, and **d** frequency of the shared and unique genera between the regions. **a**, **b**, **d** The most dominant taxa are shown. **d** Asterisks indicate a higher frequency of the genera when comparing the north and south regions

The genus *Cladosporium* was the most abundant in the south region (22%) and the second most common in the north region (12%), whereas the genus *Venturia* was the most dominant in the north (33%) (Fig. 3a). *Venturia* was abundant and found in all locations of the north region (average 32%, minimum 3% in Var, maximum 79% in Lok) but only present in two samples of the southern location Lie with a low abundance (0.4%). The south harbored 42 specific genera, among which *Podosphaera* and *Lachnum* were relatively abundant (average 8.7% and 4.3%, respectively), whereas the northern region harbored 16 specific genera with a low abundance each (< 1%).

At the family level, the northern samples harbored a higher proportion of *Venturiaceae* (genus *Venturia*), *Rhytismataceae*, and *Mycosphaerellaceae* (≥ four times higher compared to the samples from the south) (Additional file 2: Fig. S2a). The south region had a higher abundance of *Helotiaceae*, *Sclerotiniaceae*, and *Dermateaceae* (≥ two times higher compared to the north), although *Sclerotiniaceae* and *Dermateaceae* were not present in Hel and Iso of the south, respectively. *Erysiphaceae* (genus *Podosphaera*), *Hyaloscyphaceae*, *Massarinaceae* and *Cronartiaceae* were present in all southern locations but not in any of the northern locations.

At the order level, the dominant orders varied by location (Fig. 3b). Specifically, the dominant orders were *Capnodiales* in Var (north), Hel and Ves (south), *Dothideales* in Som (north) and Iso (south), *Venturiales* (genus *Venturia*) in Lok and Mal (north), and *Helotiales* in Lie (south). *Erysiphales* (genus *Podosphaera*) and *Pucciniales* were found in all locations of the south region but not in the north. *Helotiales* and *Pleosporales* were more common in the south region compared to the north (south vs north: 18% vs. 3.7% and 6% vs. 1.4%, respectively), whereas *Rhytismatales* (family *Rhytismataceae*) was more abundant in the north (11% vs. 2.4%).

*Dothideomycetes* class was dominant in both regions, and its relative abundance was higher in the north region compared to the south (81% vs. 52%). On the other hand, the abundance of *Leotiomycetes* was higher in the south region (37% vs. 17%) (Additional file 2: Fig. S2b). *Dothideomycetes* was the most dominant class in all eight locations (minimum 47%), and *Leotiomycetes* was the second abundant class (minimum 2% in Lok of the north, maximum 44% in Hel of the south). *Pucciniomycetes* (order *Pucciniales*) was found at an abundance of 6.6% in the south but not in the north region. Rare taxa were found in both regions, for example, *Microbotryomycetes*, *Exobasidiomycetes* and GS18 (phylum *Olpidiomycota*) were found only in the south with a low abundance (< 0.03%), whereas *Umbelopsidomycetes* (phylum *Mucoromycota*) was only present in the north.

### Shared and unique OTUs between two regions

There were 24 shared OTUs between the north and the south regions (Fig. 3c). In the southern samples there were 63 unique OTUs while only 28 in the north. Sixteen genera shared between regions were classified as *Cladosporium*, *Cystofilobasidium*, *Hormonema*, *Lapidomyces*, *Mycosymbioces*, *Neophaeomoniella*, *Perusta*, *Phacidium*, *Septoriella*, *Sporormiella*, *Synnemapestaloides*, *Taphrina*, *Venturia*, *Colpoma*, *Pragmopora*, *Aureobasidium* (Fig. 3d) and eight unknown genera. The north region had a higher frequency of unique OTUs that were classified as *Penicillium*, *Peniophora, Rachicladosporium,* and *Vishniacozyma*, while the south region had a higher frequency of unique OTUs, classified as e.g., *Colpoma, Pragmopora* and *Lachnum*. Three OTUs, *Hormonema macrosporum*, *Dothideales* sp., and *Cladosporium* sp., were shared between all locations. Hel of the south hosted the highest number of unique OTUs (n = 16), whereas Mal of the north had the lowest (n = 3).

### Abundance of fungal genera differed between the two regions

The genera with significantly different abundance between the north and south regions were *Venturia*, *Cladosporium* and *Podosphaera* (Additional file 2: Table S8). The genus *Venturia*, which was represented by only one species, correlated positively with the PC1 (*P* < 0.001) (Fig. 2e, Additional file 2: Table S9). *Podosphaera* and *Cladosporium* decreased with the PC1 (*P* < 0.05 and *P* < 0.001, respectively) (Additional file 2: Table S9), and *Cladosporium* had a stronger negative relationship with the PC1 compared to *Podosphaera* (Fig. 2e). The soil variables did not show significant effects in the GLMM models for the genera.

## Discussion

Bilberry fruits are among the important natural resources and economical crops in Northern Europe, but the microbial communities inhabiting inside the fruits (endophytes) are still poorly known. We explored how climatic region, weather conditions of the growth season, and soil properties affect the diversity and composition of the fungal endophytic communities in bilberry fruits collected from two different latitudinal regions in Finland. We found that (i) fungal species richness of the fruits was higher in the south, which was driven by the weather conditions of the growth season; (ii) beta diversity (variation in community structure) of the fungal communities was higher in the south and driven by the fungal richness but not by either of the measured factors, weather or soil; (iii) the weather had a small but significant effect on shaping the structure of fungal communities of bilberry fruits especially in the north; (iv) the weather strongly influenced the abundance of the fungal genera *Venturia*, *Cladosporium*, and *Podosphaera*.

### A higher diversity of fungi in the south, the weather being the driving factor of fungal richness and community structure

We found that the fungal community of bilberries in the south region had higher diversity compared to the north. Fungal richness in bilberry fruits decreased towards the north, which is in line with the research on foliar endophytic fungi by Arnold and Lutzoni [31]. In contrast to their paper, where they found a small number of classes with a large number of endophytic species in the southern latitudes, we found that samples of the south region had more diverse taxa at class to species level. The variation in fungal community structure of the south region (multivariate dispersion) was greater than that of the north, which indicates that the south had a higher beta diversity [57]. Specifically, the variation was due to the higher species richness and more unique species in each southern site.

In our study, climate (defined by climatic regions and the weather conditions of the growth season) had an impact on both the fungal richness and fungal community composition of bilberry fruits (specifically, more in the north than in the south). The climate explaining fungal composition of foliar endophytes has earlier been reported [59–63]. However, fewer reports have discovered the effect of climate on the alpha diversity of foliar endophytes. For instance, endophyte richness of leaves is reported to be associated negatively with temperature seasonality (intra annual shift in temperature) [59]. The isolation frequency of endophytes in a survey on multiple plant species was strongly linked to annual precipitation and duration of the growth season, but the endophytic alpha diversity was not [62]. In another study, foliar endophyte richness was driven by historical rainfall and temperature in *Panicum hallii* grass [60]. However, there are no previous reports showing the effect of the weather on the richness of fungal endophytes in plants, in general, or in fruits.

Vegetation growth at the high latitudes is affected primarily by the temperature and radiation [64]. Climate plays an essential role in driving vegetation diversity, species richness in particular, and total species richness hotspots occur most likely in regions with warmer growing conditions [65]. Therefore, we suggest that the warmer weather in the south region favours higher vegetation density and diversity, which could provide a richer source of fungal species for endophytes in bilberry fruits. Earlier, the source of foliar fungal endophytes was suggested to be spores emerging from dead leaves (litter) and carried by wind or water to new plants [66]. Previous studies point out that canopy tree species affected the endophytic fungal communities of the understory seedlings [67], and closed forest canopies often enhance the rate of endophyte colonization in tree leaves [30, 68]. Moreover, a recent study on foliar fungal endophytes suggested that the endophyte community structure is strongly associated with the density and composition of neighbouring plant communities [61], which could equally be the case for the fruits of dwarf shrubs, such as bilberry. These results reinforced our hypothesis that the effect of climate on the fungal richness of bilberry fruits could be due to the surrounding vegetation.

Our hypothesis was further supported by our observations in the field. Specifically, the southern sampling sites had higher plant density and plant coverage compared to the northern sites. For example, the location Lie in the south, where the average fungal richness of the fruits was the highest, had high vegetation density and coverage. The site was characterized by a diversity of trees with species such as birch, aspen, pine, Norway spruce, and undergrowth of the *Hylocomnium splendens* moss. On the other hand, the location Lok of the north, which had the lowest fungal richness, had the lowest tree density, diversity and coverage compared to the other sampling locations. This location had bilberry-dominant undercover and less abundant species, consisting mainly of crowberry, pine, spruce, and birches. This site was the only one located further away from natural parks and more recently managed with tree stumps present.

### Weather affects abundance of the fungal genera *Venturia*, *Cladosporium*, and *Podosphaera*

The north and south regions had distinguished fungal endophyte profiles at each taxonomic level. The profiles also varied between locations, although the locations in the same region resembled each other. This indicates that each region has its own signature endophyte pool. Such signature endophyte pools can be partly shaped by climate, likely via the surrounding vegetation.

At the genus level, specific taxa had significant differences in their abundances between the south and the north regions, being influenced by the weather. For example, the genus *Venturia* dominated in the three locations of the north, especially Lok, and the abundance of this genus was strongly shaped by the weather. *Venturia* spp. are well known due to many pathogenic members that cause disease in deciduous trees and can survive through the winter at the sexual stage in the litter, which becomes the main inoculum source for the next season [69]. Each species of *Venturia* has an optimal temperature and minimum hours of wetness needed for infection [69]. In our study, only one *Venturia* sp. was present and abundant in the north while rarely found in the south, which suggests that this endophytic species favours low temperatures and high precipitation and humidity of the north region for the life cycle in the bilberry fruits. The genus *Cladosporium*, however, was more abundant in the south region, potentially due to the spore season of *Cladosporium* genus being accelerated and elongated in warm and dry weather as reported by Kasprzyk et al. [70]. Similarly, some species of the *Podosphaera* genus are influenced by temperature on their sporulation and/or infection efficiency [71, 72] with their optimal temperature being 22–25 °C. We suggest that the *Podosphaera* genus in our study might favour the warmer ambient temperatures of the south, as it was present only in the southern samples.

## Conclusions

Endophytes play important roles in plants by benefiting the host in several ways, e.g., protection against pathogens or enhancing secondary metabolism [10–12]. The endophytic community also contributes to the overall health of the forests by participating in decomposition of the structural components and nutrient circulation of the litter [73]. Therefore, the richness or diversity of the endophytic community could reflect the health of the host plant and the entire forest ecosystem. Moreover, since the endophytic community can be a fingerprint linked to the geographical origin of the fruits, it becomes a potential analytical tool to trace the fruit source [17, 74]. Based on our present study, we conclude that the weather conditions of the growth season have an important impact on the richness and composition of the fungal endophytic community in bilberry fruits via the surrounding vegetation, and strongly affects the abundance of fungal taxa such as *Venturia*, *Cladosporium* and *Podosphaera*. The fungal endophytic community structure in bilberry fruits follows similar patterns as are found for foliar fungal endophytes, being shaped by various environmental factors, such as the climate and vegetation. Our study supports our earlier findings [19] on the endophytic communities in fruits of three wild berry species, suggesting that fungal endophytes of fruits mainly originate from the surrounding environment, thus being horizontally transmitted.

### Supplementary Information

**Additional file 1: Table S1.** Metadata.

**Additional file 2: Figure S1.** Pearson’s correlation values between the examined environmental variables presented by heatmap. **Figure S2.** Fungal community profiles in the two regions demonstrated by stacked bar plots at the family (**a**) and class (**b**) levels. Only the most dominant taxa are shown. **Table S2.** Explanatory variables and their description. **Table S3.** Summary of model validations and AICc values of all models. The final models are indicated in blue characters. **Table S4.** Summary of the best GLMM model of the fungal richness as a function of regions. **Table S5.** Summary of the best GLMM model of the fungal richness as a function of environmental variables. **Table S6.** Summary of the best GLMM model of the square root of distance-to-centroid values as a function of square root of richness. **Table S7.** Marginal permutation tests (9999 permutations) of the full dbRDA models with all tested environmental variables as the explanators for the community structure of the whole dataset and the north region. The dbRDA models were built for two scales: the whole dataset and the north region. **Table S8.** Summary of the differential abundance analysis performed by the ALDEx2 package. **Table S9.** Summary of the best GLMM models of the genus abundance as a function of the environmental variables.

## Data Availability

The data of this study are openly available in the ENA at EMBL-EBI (www.ebi.ac.uk) under the accession number PRJEB60612.

## References

[1] Heinonen M (2007). Antioxidant activity and antimicrobial effect of berry phenolics—a Finnish perspective. Mol Nutr Food Res.

[2] Miina J, Hotanen J-P, Salo K (2009). Modelling the abundance and temporal variation in the production of bilberry (*Vaccinium myrtillus* L.) in Finnish mineral soil forests. Silva Fennica.

[3] Zafra-Stone S, Yasmin T, Bagchi M, Chatterjee A, Vinson JA, Bagchi D (2007). Berry anthocyanins as novel antioxidants in human health and disease prevention. Mol Nutr Food Res.

[4] Manganaris GA, Goulas V, Vicente AR, Terry LA (2014). Berry antioxidants: small fruits providing large benefits. J Sci Food Agric.

[5] Trivedi P, Karppinen K, Klavins L, Kviesis J, Sundqvist P, Nguyen N (2019). Compositional and morphological analyses of wax in northern wild berry species. Food Chem..

[6] Lätti AK, Riihinen KR, Kainulainen PS (2008). Analysis of anthocyanin variation in wild populations of bilberry (*Vaccinium myrtillus* L.) in Finland. J Agric Food Chem..

[7] Jaakola L, Määttä K, Pirttilä AM, Törrönen R, Kärenlampi S, Hohtola A (2002). Expression of genes involved in anthocyanin biosynthesis in relation to anthocyanin, proanthocyanidin, and flavonol levels during bilberry fruit development. Plant Physiol.

[8] Müller DB, Vogel C, Bai Y, Vorholt JA (2016). The plant microbiota: systems-level insights and perspectives. Annu Rev Genet.

[9] Hardoim PR, van Overbeek LS, Berg G, Pirttilä AM, Compant S, Campisano A (2015). The hidden world within plants: ecological and evolutionary considerations for defining functioning of microbial endophytes. MMBR.

[10] Tejesvi MV, Segura DR, Schnorr KM, Sandvang D, Mattila S, Olsen PB (2013). An antimicrobial peptide from endophytic *Fusarium tricinctum* of *Rhododendron tomentosum* Harmaja. Fungal Divers.

[11] Gunatilaka AAL (2006). Natural products from plant-associated microorganisms: distribution, structural diversity, bioactivity, and implications of their occurrence. J Nat Prod.

[12] Koskimäki JJ, Hokkanen J, Jaakola L, Suorsa M, Tolonen A, Mattila S (2009). Flavonoid biosynthesis and degradation play a role in early defence responses of bilberry (*Vaccinium myrtillus*) against biotic stress. Eur J Plant Pathol.

[13] Redman RS, Kim YO, Woodward CJDA, Greer C, Espino L, Doty SL (2011). Increased fitness of rice plants to abiotic stress via habitat adapted symbiosis: a strategy for mitigating impacts of climate change. PLoS ONE.

[14] Baltruschat H, Fodor J, Harrach BD, Niemczyk E, Barna B, Gullner G (2008). Salt tolerance of barley induced by the root endophyte *Piriformospora indica* is associated with a strong increase in antioxidants. New Phytol.

[15] Collinge DB, Jensen B, Jørgensen HJ (2022). Fungal endophytes in plants and their relationship to plant disease. Curr Opin Microbiol.

[16] Martins F, Mina D, Pereira JA, Baptista P (2021). Endophytic fungal community structure in olive orchards with high and low incidence of olive anthracnose. Sci Rep.

[17] Francois G, Fabrice V, Didier M (2020). Traceability of fruits and vegetables. Phytochemistry.

[18] Lugtenberg BJJ, Caradus JR, Johnson LJ (2016). Fungal endophytes for sustainable crop production. FEMS Microbiol Ecol.

[19] Nguyen M-P, Lehosmaa K, Martz F, Koskimäki JJ, Pirttilä AM, Häggman H (2021). Host species shape the community structure of culturable endophytes in fruits of wild berry species (*Vaccinium myrtillus* L., *Empetrum nigrum* L. and *Vaccinium vitis-idaea* L.). FEMS Microbiol Ecol..

[20] Huang W, Cai Y, Hyde K, Corke H, Sun M (2008). Biodiversity of endophytic fungi associated with 29 traditional Chinese medicinal plants. Fungal Divers.

[21] Dong C, Wang L, Li Q, Shang Q (2021). Epiphytic and endophytic fungal communities of tomato plants. Hortic Plant J.

[22] Liu D, Howell K (2021). Community succession of the grapevine fungal microbiome in the annual growth cycle. Environ Microbiol.

[23] Gomes T, Pereira JA, Benhadi J, Lino-Neto T, Baptista P (2018). Endophytic and epiphytic phyllosphere fungal communities are shaped by different environmental factors in a mediterranean ecosystem. Microb Ecol.

[24] Dugan FM, Lupien SL, Grove GG (2002). Incidence, aggressiveness and in planta interactions of *Botrytis cinerea* and other filamentous fungi quiescent in grape berries and dormant buds in central Washington State. J Phytopathol.

[25] Tadych M, Bergen MS, Johnson-Cicalese J, Polashock JJ, Vorsa N, White JF (2012). Endophytic and pathogenic fungi of developing cranberry ovaries from flower to mature fruit: diversity and succession. Fungal Divers.

[26] Vega FE, Posada F, Catherine Aime M, Pava-Ripoll M, Infante F, Rehner SA (2008). Entomopathogenic fungal endophytes. Biol Control.

[27] Glushakova AM, Kachalkin AV (2017). Endophytic yeasts in *Malus domestica* and *Pyrus communis* fruits under anthropogenic impact. Microbiology.

[28] Li ZJ, Shen XY, Hou CL (2016). Fungal endophytes of South China blueberry (*Vaccinium dunalianum* var. *urophyllum*). Lett Appl Microbiol..

[29] Szymanski S, Longley R, Hatlen RJ, Heger L, Sharma N, Bonito G (2023). The blueberry fruit mycobiome varies by tissue type and fungicide treatment. Phytobiomes J.

[30] Arnold AE, Herre EA (2003). Canopy cover and leaf age affect colonization by tropical fungal endophytes: ecological pattern and process in *Theobroma cacao* (Malvaceae). Mycologia.

[31] Arnold AE, Lutzoni F (2007). Diversity and host range of foliar fungal endophytes: are tropical leaves biodiversity hotspots?. Ecology.

[32] R Core Team. R: a language and environment for statistical computing. Vienna: R Foundation for Statistical Computing, Austria. 2021.

[33] Hijmans RJ. raster: geographic data analysis and modeling. R package version 3.6-14.

[34] Bivand R, Keitt T, Rowlingson B. rgdal: bindings for the “geospatial” data abstraction library. R package version 1.6-4. 2023*.*

[35] Pebesma E (2018). Simple features for R: standardized support for spatial vector data. R J.

[36] Wickham H, François R, Henry L, Müller K, Vaughan D. dplyr: a grammar of data manipulation. R package version 1.1.0. 2023.

[37] Wickham H (2017). ggplot2—elegant graphics for data analysis.

[38] Muñoz Sabater J. ERA5-Land hourly data from 1981 to present. Copernicus Climate Change Service (C3S) Climate Data Store (CDS). 2019. 10.24381/cds.e2161bac. Accessed 1 May 2021.

[39] Oksanen J, Simpson GL, Blanchet FG, Kindt R, Legendre P, Minchin PR, et al. vegan: community ecology package. R package version 2.6-4. 2022.

[40] Ihrmark K, Bödeker ITM, Cruz-Martinez K, Friberg H, Kubartova A, Schenck J (2012). New primers to amplify the fungal ITS2 region—evaluation by 454-sequencing of artificial and natural communities. FEMS Microbiol Ecol.

[41] White TJ, Bruns T, Lee S, Taylor J. Amplification and direct sequencing of fungal ribosomal RNA genes for phylogenetics. In: PCR Protocols. Elsevier; 1990. p. 315–322.

[42] Bolyen E, Rideout JR, Dillon MR, Bokulich NA, Abnet CC, Al-Ghalith GA (2019). Reproducible, interactive, scalable and extensible microbiome data science using QIIME 2. Nat Biotechnol.

[43] Nguyen NH. Amplicon analysis pipeline with QIIME2. 2020. https://github.com/nnguyenlab/amplicon-pipeline. Accessed 1 Jan 2023.

[44] Bengtsson-Palme J, Ryberg M, Hartmann M, Branco S, Wang Z, Godhe A (2013). Improved software detection and extraction of ITS1 and ITS2 from ribosomal ITS sequences of fungi and other eukaryotes for analysis of environmental sequencing data. Methods Ecol Evol.

[45] Kessy UA, Allan Z, Timo P, Raivo P, Filipp I, R. Henrik N, et al. UNITE QIIME release for Fungi 2. Version 10.05.2021. UNITE Community. 2021. 10.15156/BIO/1264763.

[46] McMurdie PJ, Holmes S (2013). phyloseq: an R package for reproducible interactive analysis and graphics of microbiome census data. PLoS ONE.

[47] Nguyen NH, Smith D, Peay K, Kennedy P (2015). Parsing ecological signal from noise in next generation amplicon sequencing. New Phytol.

[48] Tedersoo L, Bahram M, Zinger L, Nilsson RH, Kennedy PG, Yang T (2022). Best practices in metabarcoding of fungi: from experimental design to results. Mol Ecol.

[49] Brooks ME, Kristensen K, Van Benthem KJ, Magnusson A, Berg CW, Nielsen A (2017). glmmTMB balances speed and flexibility among packages for zero-inflated generalized linear mixed modeling. R J.

[50] Hartig F. DHARMa: residual diagnostics for hierarchical (multi-level / mixed) regression models. R package version 0.4.6. 2022.

[51] Bartoń K. MuMIn: multi-model inference. R package version 1.47.1. 2022.

[52] Lahti L, Shetty S. microbiome R package. 2019.

[53] Liu C, Cui Y, Li X, Yao M (2021). *microeco*: an R package for data mining in microbial community ecology. FEMS Microbiol Ecol..

[54] Fernandes AD, Reid JNS, Macklaim JM, McMurrough TA, Edgell DR, Gloor GB (2014). Unifying the analysis of high-throughput sequencing datasets: Characterizing RNA-seq, 16S rRNA gene sequencing and selective growth experiments by compositional data analysis. Microbiome.

[55] Kassambara A. ggpubr: “ggplot2” based publication ready plots. R package version 0.6.0. 2023.

[56] Dormann CF, Elith J, Bacher S, Buchmann C, Carl G, Carré G (2013). Collinearity: a review of methods to deal with it and a simulation study evaluating their performance. Ecography.

[57] Anderson MJ (2006). Distance-based tests for homogeneity of multivariate dispersions. Biometrics.

[58] Torondel B, Ensink JHJ, Gundogdu O, Ijaz UZ, Parkhill J, Abdelahi F (2016). Assessment of the influence of intrinsic environmental and geographical factors on the bacterial ecology of pit latrines. Microb Biotechnol.

[59] Oita S, Ibáñez A, Lutzoni F, Miadlikowska J, Geml J, Lewis LA (2021). Climate and seasonality drive the richness and composition of tropical fungal endophytes at a landscape scale. Commun Biol.

[60] Giauque H, Hawkes CV (2016). Historical and current climate drive spatial and temporal patterns in fungal endophyte diversity. Fungal Ecol.

[61] Darcy JL, Swift SOI, Cobian GM, Zahn GL, Perry BA, Amend AS (2020). Fungal communities living within leaves of native Hawaiian dicots are structured by landscape-scale variables as well as by host plants. Mol Ecol.

[62] U’ren JM, Lutzoni F, Miadlikowska J, Laetsch AD, Arnold AE (2012). Host and geographic structure of endophytic and endolichenic fungi at a continental scale. Am J Bot.

[63] Zimmerman NB, Vitousek PM (2012). Fungal endophyte communities reflect environmental structuring across a Hawaiian landscape. Proc Natl Acad Sci U S A.

[64] Wu D, Zhao X, Liang S, Zhou T, Huang K, Tang B (2015). Time-lag effects of global vegetation responses to climate change. Glob Change Biol.

[65] Niskanen AKJ, Heikkinen RK, Väre H, Luoto M (2017). Drivers of high-latitude plant diversity hotspots and their congruence. Biol Conserv.

[66] Herre EA, Mejía LC, Kyllo DA, Rojas E, Maynard Z, Butler A (2007). Ecological implications of anti-pathogen effects of tropical fungal endophytes and mycorrhizae. Ecology.

[67] Diversity Kato S, Fukasawa Y, Seiwa K (2017). Canopy tree species and openness affect foliar endophytic fungal communities of understory seedlings. Ecol Res.

[68] Petrini O, Carroll G (1981). Endophytic fungi in foliage of some Cupressaceae in Oregon. Can J Bot.

[69] González-Domínguez E, Armengol J, Rossi V (2017). Biology and epidemiology of *Venturia* species affecting fruit crops: a review. Front Plant Sci.

[70] Kasprzyk I, Kaszewski BM, Weryszko-Chmielewska E, Nowak M, Sulborska A, Kaczmarek J (2016). Warm and dry weather accelerates and elongates *Cladosporium* spore seasons in Poland. Aerobiologia (Bologna).

[71] Sombardier A, Savary S, Blancard D, Jolivet J, Willocquet L (2009). Effects of leaf surface and temperature on monocyclic processes in *Podosphaera aphanis*, causing powdery mildew of strawberry. Can J Plant Pathol.

[72] Peetz AB, Mahaffee WF, Gent DH (2009). Effect of temperature on sporulation and infectivity of *Podosphaera macularis* on *Humulus lupulus*. Plant Dis.

[73] Osono T (2006). Role of phyllosphere fungi of forest trees in the development of decomposer fungal communities and decomposition processes of leaf litter. Can J Microbiol.

[74] El Sheikha AF, Condur A, Métayer I, Le Nguyen DD, Loiseau G, Montet D (2009). Determination of fruit origin by using 26S rDNA fingerprinting of yeast communities by PCR-DGGE: preliminary application to *Physalis* fruits from Egypt. Yeast.

