# Supplementary material for: Weather in two climatic regions shapes the diversity and drives the structure of fungal endophytic community of bilberry (Vaccinium myrtillus L.) fruit
Source: Environ Microbiome. 2024 Jan 22;19:7. doi: 10.1186/s40793-024-00551-y (PMC10802051; doi:10.1186/s40793-024-00551-y)
Supplement: Supplementary file 2 — Additional file 2: Figure S1. Pearson’s correlation values between the examined environmental variables presented by heatmap. Figure S2. Fungal community profiles in the two regions demonstrated by stacked bar plots at the family (a) and class (b) levels. Only the most dominant taxa are shown. Table S2. Explanatory variables and their description. Table S3. Summary of model validations and AICc values of all models. The final models are indicated in blue characters. Table S4. Summary of the best GLMM model of the fungal richness as a function of regions. Table S5. Summary of the best GLMM model of the fungal richness as a function of environmental variables. Table S6. Summary of the best GLMM model of the square root of distance-to-centroid values as a function of square root of richness. Table S7. Marginal permutation tests (9999 permutations) of the full dbRDA models with all tested environmental variables as the explanators for the community structure of the whole dataset and the north region. The dbRDA models were built for two scales: the whole dataset and the north region. Table S8. Summary of the differential abundance analysis performed by the ALDEx2 package. Table S9. Summary of the best GLMM models of the genus abundance as a function of the environmental variables. [file 40793_2024_551_MOESM2_ESM.docx]

**Fig. S1** Pearson’s correlation values between the examined environmental variables presented by heatmap.


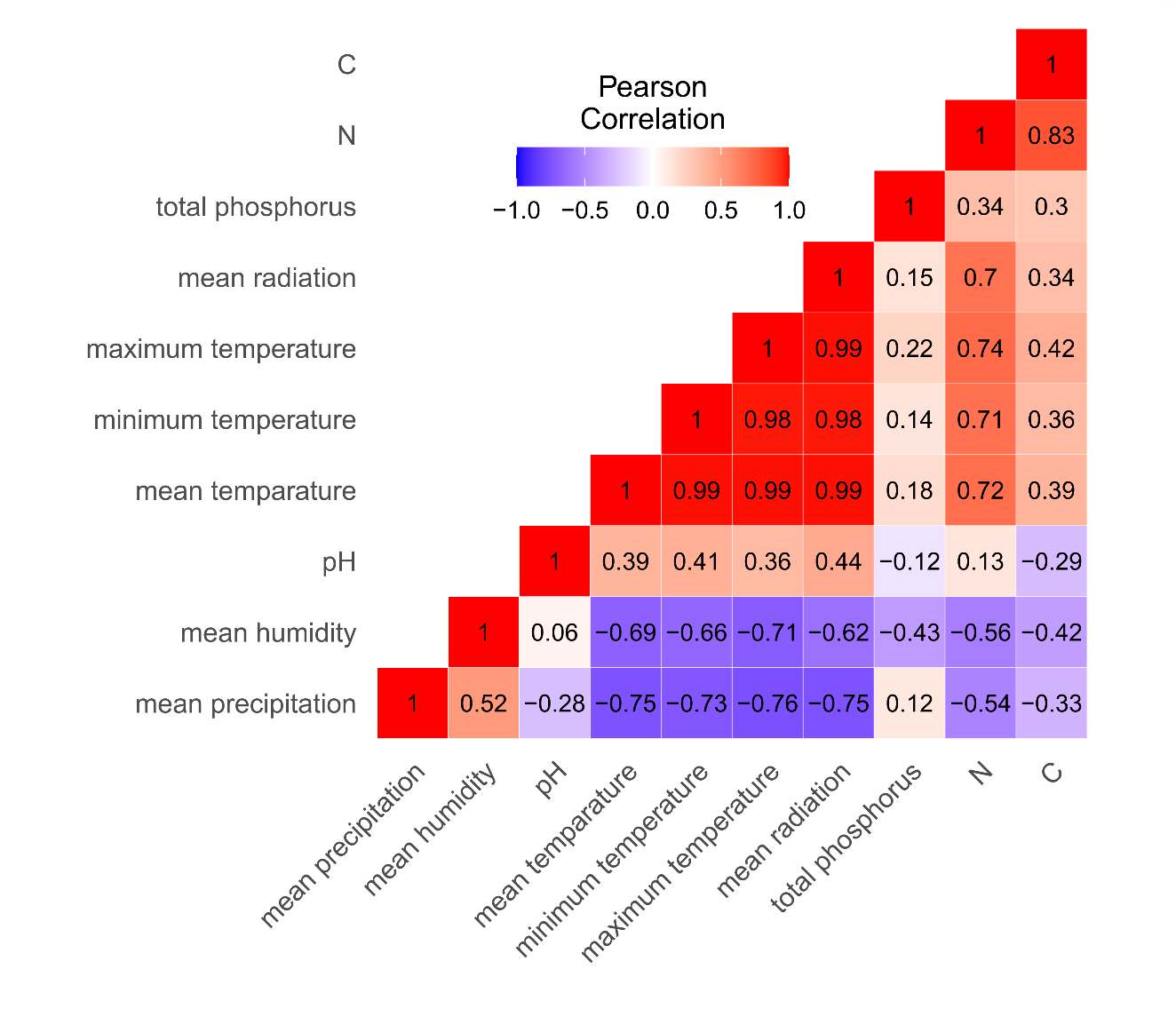


**Fig. S2** Fungal community profiles in the two regions demonstrated by stacked bar plots at the family (A) and class (B) levels. Only the most dominant taxa are shown.


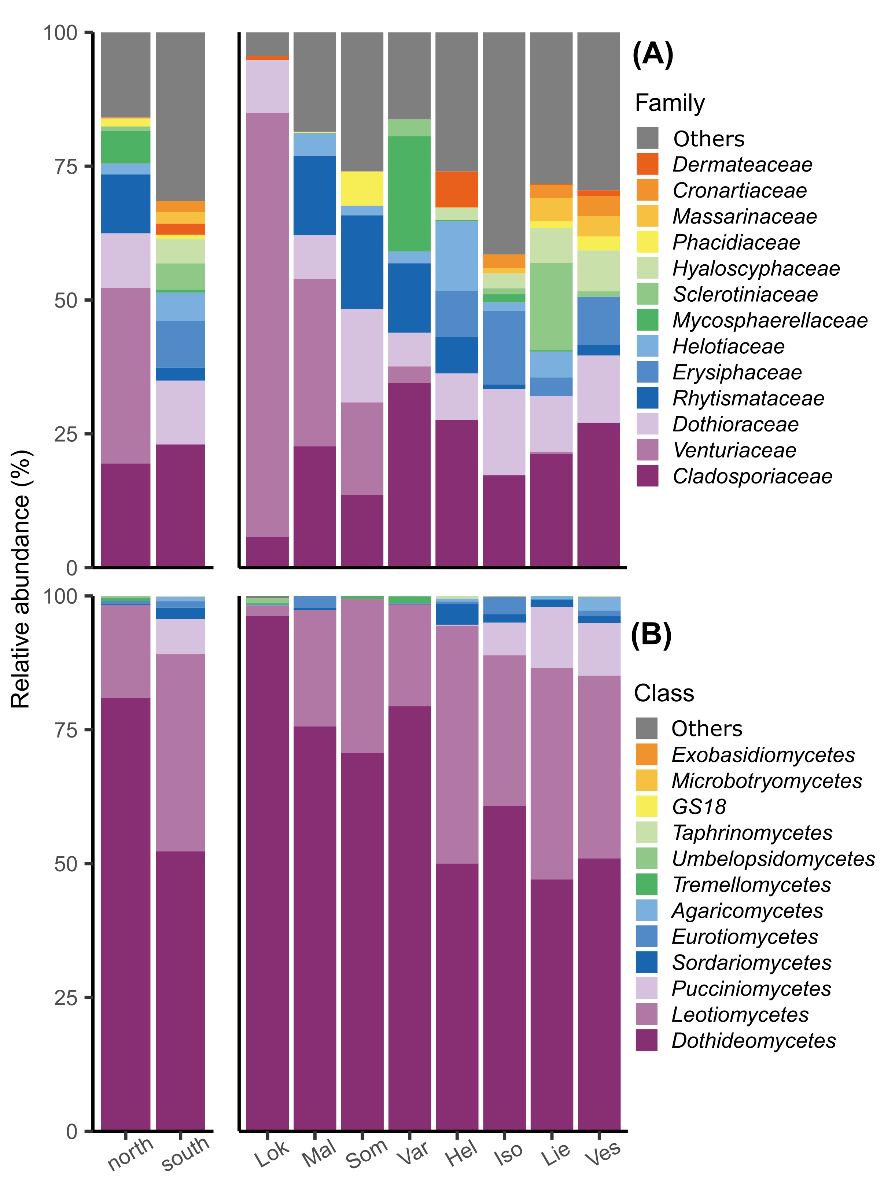


**Table S1** metadata (in a separate excel file)

**Table S2** Explanatory variables and their description

|  | **VARIABLE** | **DESCRIPTION** |
| --- | --- | --- |
| **WEATHER** | Mean temperature |  |
|  | Maximum temperature |  |
|  | Minimum temperature |  |
|  | Mean precipitation |  |
|  | Mean humidity |  |
|  | Mean radiation |  |
| **TOPOGRAPHY** | Altitude |  |
| **PC1** | PC1 | PC1 axis of PCA for weather variables |
| **SOIL** | C | Carbon content |
|  | N | Nitrogen content |
|  | TP | Total phosphorus |
|  | pH |  |
|  | CN | C:N ratio |
| **FACTOR** | Region |  |
|  | Location |  |
|  | Clone (random) |  |
| **COVARIATE** | Sequencing depth |  |
|  | Vegetation type |  |

**Table S3** Summary of model validations and AICc values of all models. The final models are indicated in blue characters.

| **Model** | **AICc** | **Model validation** |
| --- | --- | --- |
| **Response variable: fungal richness \| explanatory variable: region** |  |  |
| m1 <- glmmTMB (richness ~ region + offset (log sequencing depth) + vegetation type + (1\|region:clone), data = metadata, family = poisson(), REML = T) | 560.0474 | pass |
| m1.1 <- glmmTMB (richness ~ region + offset (log sequencing depth) + (1\|region:clone), data = metadata, family = poisson(), REML = T) | 554.6011 | fail |
| **Response variable: fungal richness \| explanatory variable: environmental variables** |  |  |
| m2 <- glmmTMB (richness ~ PC1 + CN + TP + pH + offset (log sequencing depth) + vegetation type + (1\|region:clone), data = metadata, family = poisson(), REML = T) | 572.9129 | pass |
| m2.1 <- glmmTMB (richness ~ PC1 + CN + TP + pH + offset (log sequencing depth) + (1\|region:clone), data = metadata, family = poisson(), REML = T) | 567.1198 | fail |
| m2.2 <- glmmTMB (richness ~ PC1 + TP + pH + offset (log sequencing depth) + (1\|region:clone), data = metadata, family = poisson(), REML = T) | 563.1118 | fail |
| m2.3 <- glmmTMB (richness ~ PC1 + TP + offset (log sequencing depth) + (1\|region:clone), data = metadata, family = poisson(), REML = T) | 559.2997 | pass |
| m2.4 <- glmmTMB (richness ~ PC1 + offset (log sequencing depth) + (1\|region:clone), data = metadata, family = poisson(), REML = T) | 556.2189 | fail |
| **Response variable: fungal richness \| explanatory variable: environmental variables \| for each region** |  |  |
| m.north <- glmmTMB (richness ~ PC1 + CN + TP + pH + offset (log sequencing depth) + vegetation type + (1\|region:clone), data = metadata north, family = poisson(), REML = T) | 267.5424 | pass |
| m.south <- glmmTMB (richness ~ PC1 + CN + TP + pH + offset (log sequencing depth) + vegetation type + (1\|region:clone), data = metadata south, family = poisson(), REML = T) | 321.3638 | pass |
| **Response variable: distance-to-centroid \| explanatory variable: environmental variables** |  |  |
| m3 <- glmmTMB (sqrt (distance to centroid) ~ PC1 + CN + TP + pH + offset (log sequencing depth) + vegetation type + (1\|region:clone), data = metadata, family = gaussian(), REML = T) | 230.7039 | pass |
| **Response variable: distance-to-centroid \| explanatory variable: fungal richness** |  |  |
| m7 <- glmmTMB (sqrt (distance to centroid) ~ sqrt (richness) + vegetation type + offset (log sequencing depth) + (1\|region:clone), data = metadata, family = gaussian(), REML = T) | 147.3237 | pass |
| m7.1 <- glmmTMB (sqrt (distance to centroid) ~ sqrt (richness) + offset (log sequencing depth) + (1\|region:clone), data = metadata, family = gaussian(), REML = T) | 137.8882 | pass |
| **Response variable: *Podosphaera* abundance \| explanatory variable: environmental variables** |  |  |
| m4 <- glmmTMB (abundance ~ PC1 + CN + TP + pH + offset (log sequencing depth) + vegetation type + (1\|region:clone), data = metadata, family = nbinom2(), REML = F) | NA | pass |
| m4.1 <- glmmTMB (abundance ~ PC1 + CN + TP + pH + offset (log sequencing depth) + (1\|region:clone), data = metadata, family = nbinom2(), REML = F) | 292.1874 | pass |
| m4.2 <- glmmTMB (abundance ~ PC1 + TP + pH + offset (log sequencing depth) + (1\|region:clone), data = metadata, family = nbinom2(), REML = F) | 290.3283 | pass |
| m4.3 <- glmmTMB (abundance ~ PC1 + pH + offset (log sequencing depth) + (1\|region:clone), data = metadata, family = nbinom2(), REML = F) | 288.4245 | pass |
| m4.4 <- glmmTMB (abundance ~ PC1 + offset (log sequencing depth) + (1\|region:clone), data = metadata, family = nbinom2(), REML = F) | 287.2086 | fail |
| **Response variable: *Venturia* abundance \| explanatory variable: environmental variables** |  |  |
| m5 <- glmmTMB (abundance ~ PC1 + CN + TP + pH + offset (log sequencing depth) + vegetation type + (1\|region:clone), data = metadata, family = poisson(), REML = F) | 2663.501 | pass |
| m5.1 <- glmmTMB (abundance ~ PC1 + CN + TP + pH + offset (log sequencing depth) + (1\|region:clone), data = metadata, family = poisson(), REML = F) | 2659.192 | fail |
| m5.2 <- glmmTMB (abundance ~ PC1 + TP + CN + offset (log sequencing depth) + (1\|region:clone), data = metadata, family = poisson(), REML = F) | 2657.372 | pass |
| m5.3 <- glmmTMB (abundance ~ PC1 + TP + offset (log sequencing depth) + (1\|region:clone), data = metadata, family = poisson(), REML = F) | 2655.299 | pass |
| m5.4 <- glmmTMB (abundance ~ PC1 + offset (log sequencing depth) + (1\|region:clone), data = metadata, family = nbinom2(), REML = F) | 2653.525 | fail |
| **Response variable: *Cladosporium* abundance \| explanatory variable: environmental variables** |  |  |
| m6 <- glmmTMB (abundance ~ PC1 + CN + TP + pH + offset (log sequencing depth) + vegetation type + (1\|region:clone), data = metadata, family = nbinom1(), REML = F) | 950.3769 | pass |
| m6.1 <- glmmTMB (abundance ~ PC1 + CN + TP + pH + offset (log sequencing depth) + (1\|region:clone), data = metadata, family = nbinom1(), REML = F) | 946.9042 | pass |
| m6.2 <- glmmTMB (abundance ~ PC1 + CN + TP + offset (log sequencing depth) + (1\|region:clone), data = metadata, family = nbinom1(), REML = F) | 944.6919 | pass |
| m6.3 <- glmmTMB (abundance ~ PC1 + TP + offset (log sequencing depth) + (1\|region:clone), data = metadata, family = nbinom1(), REML = F) | 942.5431 | pass |
| m6.4 <- glmmTMB (abundance ~ PC1 + offset (log sequencing depth) + (1\|region:clone), data = metadata, family = nbinom1(), REML = F) | 941.4011 | pass |

**Table S4** Summary of the best GLMM model of the fungal richness as a function of regions. Predictor variables included in the model are denoted in the Fixed effects column. ns: non-significant (*P* > 0.1).

| Fixed effects | Estimate | Std. Error | z value | *P* |
| --- | --- | --- | --- | --- |
| (Intercept) | -10.167 | 0.127 | -79.970 | < 0.001 |
| south | 0.373 | 0.189 | 1.970 | < 0.05 |
| Vegetation type: mixed forest on mineral soil | 0.075 | 0.306 | 0.240 | ns |
| Vegetation type: mixed forest on peatland | 0.391 | 0.468 | 0.840 | ns |
| Vegetation type: peatbogs | -0.161 | 0.545 | -0.300 | ns |

**Table S5** Summary of the best GLMM model of the fungal richness as a function of environmental variables. Predictor variables included in the model are denoted in the Fixed effects column. ns: non-significant (*P* > 0.1).

| Fixed effects | Estimate | Std. Error | z value | *P* |
| --- | --- | --- | --- | --- |
| (Intercept) | -10.0 | 0.1 | -122.5 | <0.001 |
| PC1 | -0.2 | 0.1 | -2.3 | <0.05 |
| TP | 0.1 | 0.1 | 1.5 | ns |

**Table S6** Summary of the best GLMM model of the square root of distance-to-centroid values as a function of square root of richness. Predictor variables included in the model are denoted in the Fixed effects column. ns: non-significant (*P* > 0.1).

| Fixed effects | Estimate | Std. Error | z value | *P* |
| --- | --- | --- | --- | --- |
| (Intercept) | -11.4 | 0.1 | -78.6 | <0.001 |
| sqrt(richness) | 0.6 | 0.1 | 10.3 | <0.001 |

**Table S7** Marginal permutation tests (9999 permutations) of the full dbRDA models with all tested environmental variables as the explanators for the community structure of the whole dataset and the north region. The dbRDA models were built for two scales: the whole dataset and the north region. Df: degrees of freedom. ns: non-significant (*P* > 0.1). Significant terms are in bold.

|  | *Whole dataset* | | | |  | *North region* | | | |
| --- | --- | --- | --- | --- | --- | --- | --- | --- | --- |
|  | Df | Variance | *F* | *P* |  | Df | Variance | *F* | *P* |
| **PC1** | **1** | **0.26** | **2.87** | **< 0.01** |  | **1** | **0.41** | **3.26** | **< 0.05** |
| Total phosphorus | 1 | 0.09 | 1.05 | ns |  | 1 | 0.17 | 1.35 | ns |
| C:N ratio | 1 | 0.08 | 0.89 | ns |  | 1 | 0.11 | 0.88 | ns |
| pH | 1 | 0.14 | 1.59 | ns |  | 1 | 0.15 | 1.17 | ns |
| Residual | 100 | 9.01 |  |  |  | 49 | 6.16 |  |  |

**Table S8** Summary of the differential abundance analysis performed by the ALDEx2 package

| Taxon | adjusted *P* | median effect size | overlap |
| --- | --- | --- | --- |
| *Podosphaera* | <0.05 | 0.32 | 0.35 |
| *Venturia* | <0.001 | -1.17 | 0.11 |
| *Cladosporium* | <0.05 | 0.37 | 0.34 |

**Table S9** Summary of the best GLMM models of the genus abundance as a function of the environmental variables. Predictor variables included in the model are denoted in the Fixed effects column. ns: non-significant (*P* > 0.1).

| *Venturia* | | | | |
| --- | --- | --- | --- | --- |
| Fixed effects | Estimate | Std. Error | z value | *P* |
| (Intercept) | -13.1 | 0.9 | -14.9 | <0.001 |
| PC1 | 4.1 | 0.8 | 5.0 | <0.001 |
| TP | -0.4 | 0.7 | -0.6 | ns |

| *Podosphaera* | | | | |
| --- | --- | --- | --- | --- |
| Fixed effects | Estimate | Std. Error | z value | *P* |
| (Intercept) | -14.0 | 2.6 | -5.4 | <0.001 |
| PC1 | -6.1 | 2.7 | -2.3 | <0.05 |
| pH | -0.6 | 0.5 | -1.0 | ns |

| *Cladosporium* | | | | |
| --- | --- | --- | --- | --- |
| Fixed effects | Estimate | Std. Error | z value | *P* |
| (Intercept) | -5.2 | 0.3 | -16.1 | <0.001 |
| PC1 | -0.5 | 0.1 | -3.4 | <0.001 |
